# Induction of Type I Interferon Signaling Determines the Relative Pathogenicity of *Staphylococcus aureus* Strains

**DOI:** 10.1371/journal.ppat.1003951

**Published:** 2014-02-20

**Authors:** Dane Parker, Paul J. Planet, Grace Soong, Apurva Narechania, Alice Prince

**Affiliations:** 1 Department of Pediatrics, Columbia University, New York, New York, United States of America; 2 Sackler Institute for Comparative Genomics, American Museum of Natural History, New York, New York, United States of America; University of Nebraska Medical Center, United States of America

## Abstract

The tremendous success of *S. aureus* as a human pathogen has been explained primarily by its array of virulence factors that enable the organism to evade host immunity. Perhaps equally important, but less well understood, is the importance of the intensity of the host response in determining the extent of pathology induced by *S. aureus* infection, particularly in the pathogenesis of pneumonia. We compared the pathogenesis of infection caused by two phylogenetically and epidemiologically distinct strains of *S. aureus* whose behavior in humans has been well characterized. Induction of the type I IFN cascade by strain 502A, due to a NOD2-IRF5 pathway, was the major factor in causing severe pneumonia and death in a murine model of pneumonia and was associated with autolysis and release of peptidogylcan. In contrast to USA300, 502A was readily eliminated from epithelial surfaces in vitro. Nonetheless, 502A caused significantly increased tissue damage due to the organisms that were able to invade systemically and trigger type I IFN responses, and this was ameliorated in *Ifnar* ^-/-^ mice. The success of USA300 to cause invasive infection appears to depend upon its resistance to eradication from epithelial surfaces, but not production of specific toxins. Our studies illustrate the important and highly variable role of type I IFN signaling within a species and suggest that targeted immunomodulation of specific innate immune signaling cascades may be useful to prevent the excessive morbidity associated with *S. aureus* pneumonia.

## Author Summary

The virulence of the *S. aureus*, and especially that associated with the epidemic MRSA USA300 strains, has been primarily explained by its expression of a specific set of virulence factors. We postulated that that intensity of the host innate immune response activated by specific staphylococcal strains is also critical in determining the severity of infection. By comparing two divergent *S. aureus* strains, USA300, associated with invasive disease, and 502A, primarily associated with surface colonization, we found that virulence was directly associated with the ability of a strain to stimulate the type I interferon pathway. The morbidity and mortality associated with USA300 could be attributed to its resistance to killing at mucosal surfaces; once past the epithelial barrier it caused less pathology than 502A. In contrast, although 502A was more readily killed by epithelial antimicrobial factors, once into the lung, it caused significantly more pathology in a mouse model of acute pneumonia. These observations suggest that modulating the host response to infection may be useful in preventing some of the disease associated with *S. aureus* pneumonia.

## Introduction

*Staphylococcus aureus* is an important pathogen causing skin and soft tissue infections as well as pneumonia and superinfection post influenza [1]. The development of antibiotic resistance, in particular methicillin resistant *S. aureus* (MRSA) and the highly transmissible clone USA300 are of significant concern [2], [3]. While USA300 strains can colonize humans asymptomatically, they can lead to invasive infections, causing substantial morbidity and mortality [4], [5]. It is now practice to de-colonize patients in hospital settings to prevent infection [6]. The success of *S. aureus* is greatly attributed to its expression of multiple virulence factors however, the inflammatory response is a major component of the host pathology produced by infection. Multiple cell types in the lung participate in recognition and acute response to infection such as epithelial cells, macrophages, neutrophils and dendritic cells [7]. These cells in turn activate numerous signaling cascades in response to *S. aureus* PAMPs in the airway and these overlapping responses can contribute to pathology [8].

One host pathway activated upon *S. aureus* infection is the type I IFN pathway [9], [10]. Although initially identified for its role in viral infections type I IFN signaling is activated by bacteria via several different intracellular and cytosolic receptors [11], [12]. *S. aureus* USA300 activates this pathway via TLR9 recognition of DNA [10]. Activation of the pathway leads to production of IFN- β that binds to its cognate receptor interferon alpha/beta receptor, IFNAR, leading to downstream gene products [13]. To determine the impact of specific human innate immune responses on the pathogenesis of invasive *S. aureus* infection, we compared two divergent strains of *S. aureus*; the MRSA strain USA300 that is currently epidemic in the USA and the penicillin susceptible 502A, once considered as non-pathogenic [14].

Strain 502A was used extensively in the 1960's in bacterial interference studies, whereby its colonization of newborns protected them against the virulent circulating strain [14]–[16]. Thousands of infants were effectively colonized via nares and umbilicus with 502A and it was later shown adults could also be colonized and protected against invasive strains [17]. 502A was able to persist for months, rarely causing infection. However, some studies did point to localized infections and one case of sepsis was reported [18], [19]. Even though 502A displayed great benefit, to our knowledge no studies were undertaken to understand why it was not invasive or the mechanism(s) associated with its only occasional cases of disease.

We were interested if differential sensing by the host contributed to the disparate pathogenicity of *S. aureus* USA300 and 502A, why is USA300 associated with local or systemic infection in colonized hosts while 502A for the most part remains superficial and is not virulent. The distinctive responses of USA300 and 502A could be due to individual virulence factors; alternatively the nature of the host response could explain their mechanisms of pathogenesis. We show that a distinguishing characteristic of USA300 is its ability to invade into to cross the mucosal barrier and its ability to cause pathology beyond this barrier is actually less than the non-invasive 502A strain. We show herein that the non-invasive *S. aureus* strain 502A leads to differential type I IFN signaling. This type I IFN response is activated by uptake of live bacteria that signal via NOD2 and IRF5, in contrast to USA300. In a model of acute pneumonia 502A unexpectedly causes significantly more pathology and disease that is the result of host type I IFN signaling.

## Results

### 502A is less invasive than USA300

**Figure 1:**
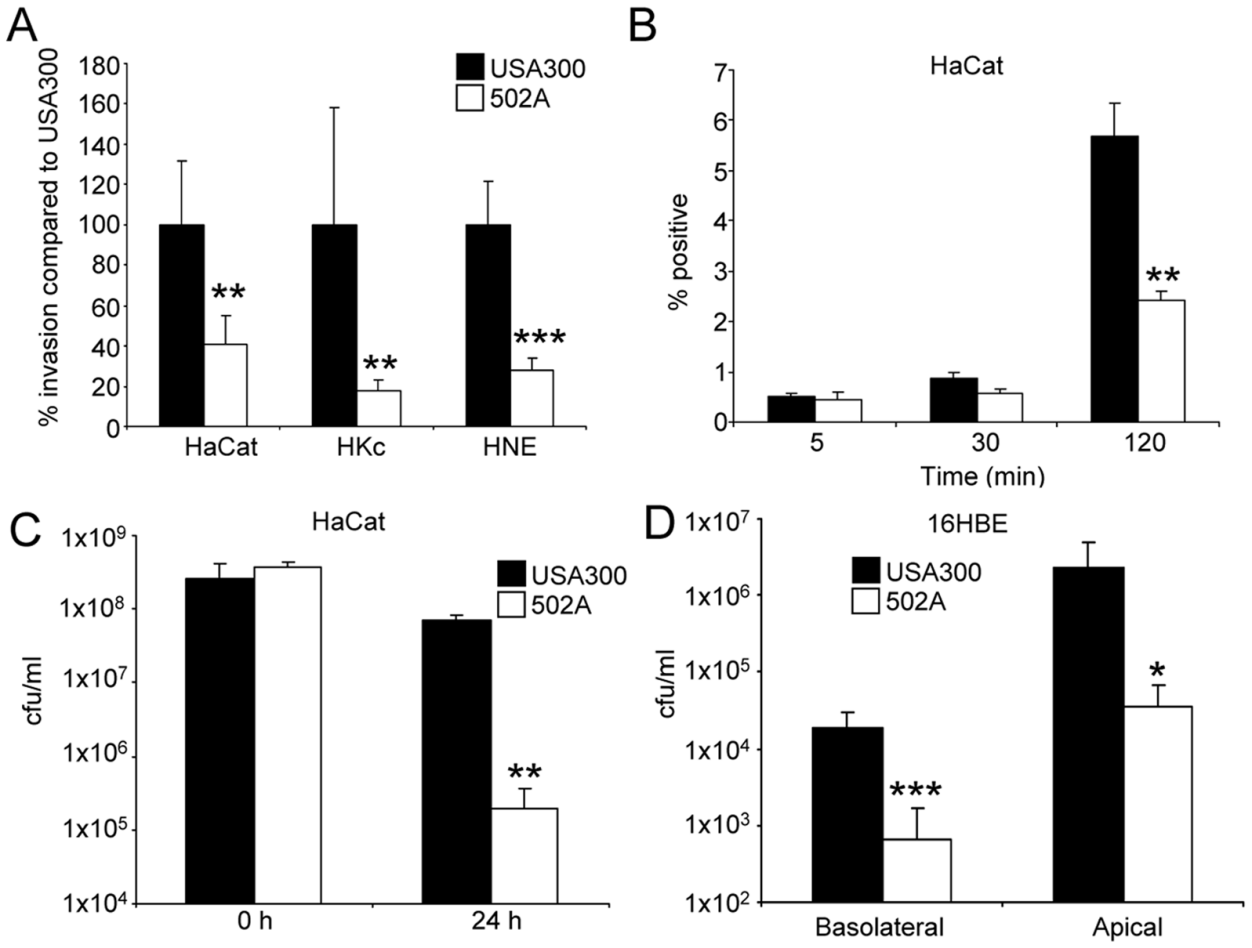
*S. aureus* 502A is less invasive than USA300. A) Invasiveness of USA300 and 502A was assessed by gentamicin protection assays in HaCat, human keratinocyte (HKc) and human nasal epithelial cells (HNE). Data is presented as a percentage of USA300 bacterial counts (n = 6). B) Uptake of fluorescently labeled *S. aureus* into HaCat cells. n = 3. C) Transmigration assay of USA300 and 502A. 5×10^7^ cfu of each strain was applied to the apical side of polarized 16HBE airway epithelial cells for 24 h before bacteria were enumerated in the basolateral and apical compartments (n = 16). D) Spent 24 h HaCat culture supernatants were incubated with 2×10^7^ cfu/ml of USA300 or 502A for 24 h before bacteria were enumerated. Data are from at least two independent experiments. ***P<0.001, **P<0.01 and *P<0.05 compared to USA300.

While both USA300 and 502A are capable of colonizing humans, USA300, unlike 502A, is highly invasive [5], [14], [20]. We directly compared their relative ability to penetrate mucosal barriers. Comparison of 502A to USA300 in gentamicin protection assays of several primary and immortalized skin cells lines showed 502A to have reduced invasiveness. Invasion of 502A was 60% less than USA300 in human keratinocyte HaCat cells (P<0.01), 83% less in primary human keratinocytes (P<0.01) and 73% less in human nasal epithelial cells (P<0.001) (Figure 1A). The reduced invasiveness of 502A was not due to host-derived killing as uptake of 502A was equivalent at early time points compared to USA300 (Figure 1B) but by 2 h 57% less HaCat cells had 502A inside of them (Figure 1B). Invasiveness of both strains was further tested by incubation with polarized airway epithelial cells and assessing their ability to migrate across the epithelial barrier. While USA300 transmigrated on average at levels of 1.8×10^4^ cfu to the basolateral compartment, 502A was over 27-fold less at 6.6×10^2^ cfu, on average (Figure 1C).

We hypothesized that the reduced invasiveness of 502A might be due to increased susceptibility to cell wall targeted killing. We found that 502A bacterial numbers remaining in the apical compartment from polarized 16HBE transmigration experiments were reduced by over 63-fold (P<0.05) compared to USA300 (Figure 1C). Similarly, incubation of 502A with spent media from the HaCat human keratinocyte line led to a 27-fold reduction (P<0.01) in 502A numbers compared to USA300 (Figure 1D). These data indicate that 502A is less invasive and is unable to cross the epithelial barrier, entirely consistent with the clinical data showing remarkably little invasion despite high inocula given to neonates.

### The genome of 502A contains similar virulence potential to USA300

**Figure 2:**
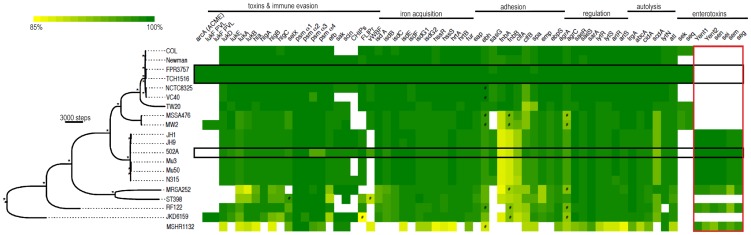
Whole genome phylogeny and heat map of gene content. The phylogenetic tree was constructed using whole genome alignment of gene coding regions for the strains pictured using the maximum parsimony (MP) optimality criterion (32,097 parsimony informative characters). Branch lengths are proportional to the number of nucleotide changes inferred for each branch. * denotes branches with 100% bootstrap support. The MP tree length was 74,526 steps (rescaled consistency index = 0.658). The staphylococcal genome MSH1132 was used as an outgroup. The heat map shows percent nucleotide identity over the entire sequence length based on BLAST comparison to each representative gene from the *S. aureus* TCH1516 genome as a query sequence. The area boxed in red shows comparisons using *S. aureus* 502A genes as queries. A cutoff of >84% nucleotide identity was used to determine presence of each gene. # denotes BLAST alignments of less than 95% of the sequence gene length. USA300 strains (FPR3757 and TCH1516) and 502A are highlighted in black boxes.

To gain insight into the disparate invasive properties of USA300 and 502A we sequenced the genome of 502A. Phylogenetic analysis using concatenated genome data shows 502A to be genetically distinct from USA300, more closely related to the MRSA strains JH1, JH9, Mu3, Mu50 and N315 (Figure 2) [21], [22]. Analysis of virulence factor composition between the two strains revealed a high level of conservation at both the gene and nucleotide level (Figure 2). All the major virulence factors including α-toxin, leukocidins, adhesion factors, protein A, iron acquisition genes, phenol soluble modulins and regulatory factors were present. We did not observe differences in protein level for α-toxin while there was a decrease in protein A (Figure S1). At the nucleotide level there was a high level of identity, the exception was fibronectin binding protein A and B with 85% nucleotide identity between USA300 and 502A. One difference was the enterotoxin complement of genes, USA300 encodes *sek* and *seq* while 502A encoded several including *seg*, *sei*, *sem* and *sen* (Figure 2). Consistent with 502A not being a MRSA or USA300 strain, it did not encode for the Panton-Valentine leukocidin or the arginine catabolic mobile element (ACME) [2] that has been shown to aid survival in the acidic environment found on the skin [23], [24]. Thus, analysis of the genome of 502A indicated it possesses many of the virulence factors to cause disease.

### 502A displays enhanced virulence in a murine pneumonia model

Although 502A was used extensively in infants to protect against infection from other more virulent *S. aureus* strains prevalent at the time [14], [15], there were several cases of local infection and septicemia as a result of 502A inoculation [18], [19]. We therefore investigated the ability of 502A to cause infection when the mucosal barrier is bypassed, in a model of acute murine pneumonia.

**Figure 3:**
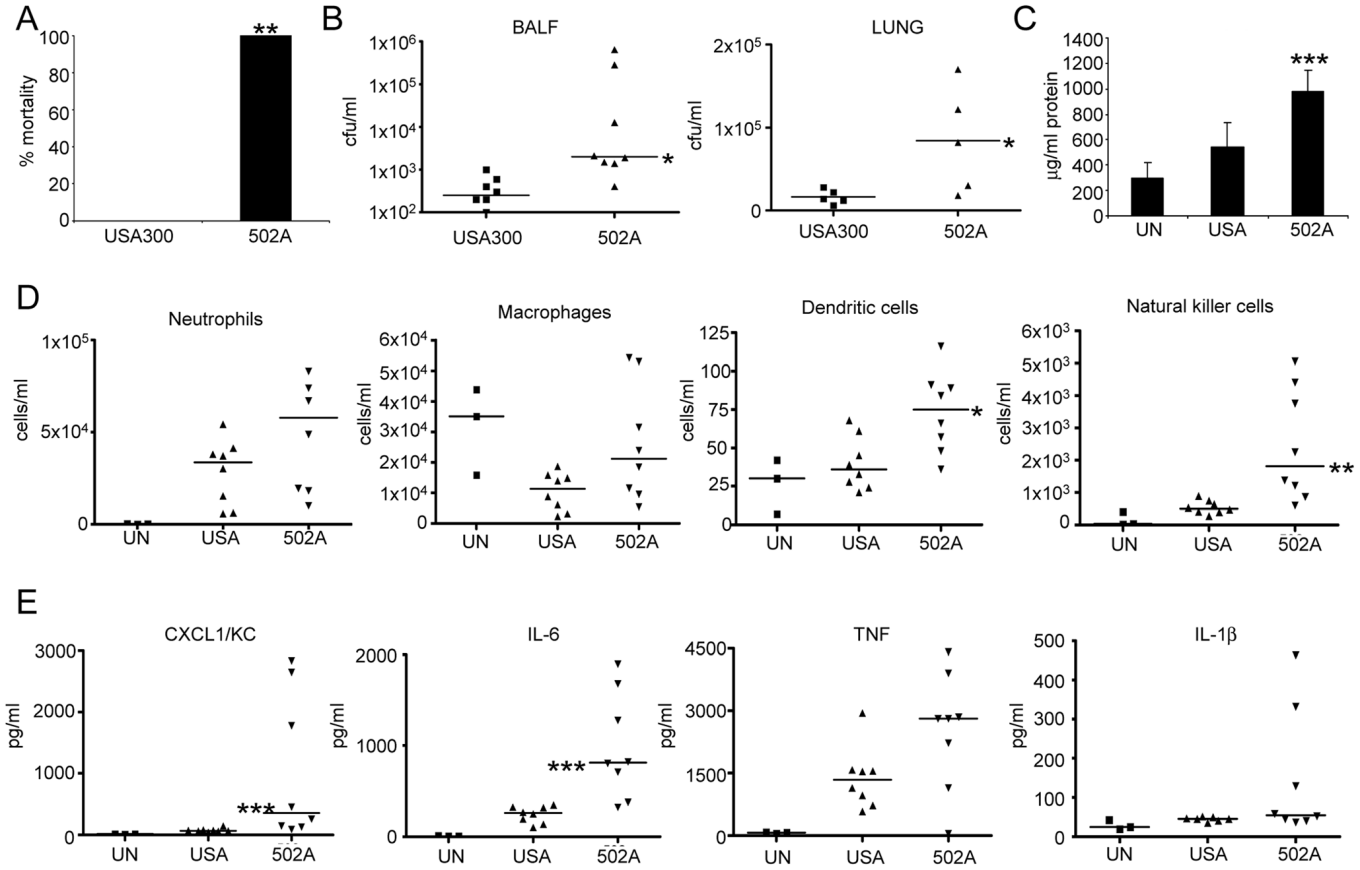
502A displays increased virulence in a murine model of acute pneumonia. A) *S. aureus* USA300 and 502A were infected intranasally (10^8^ cfu) for 20 h in C57Bl/6J mice and mortality assessed (n = 5). WT C57Bl/6J mice were infected with 10^7^ cfu of each strain for 20 h. B) BALF and lung homogenate were assessed for bacterial numbers. C) Total protein content in BALF (n = 8). D) Cells in BALF were stained with fluorescent antibodies and analyzed by flow cytometry. E) Cytokines were measure by ELISA from BALF. Data are from at least two independent experiments. Each point represents a mouse. Lines display median values. ***P<0.001, **P<0.01 and *P<0.05, compared to WT infected controls.

At an inoculum that leads to zero mortality in mice with USA300 (7×10^7^ cfu), mortality with 502A was 100% (P<0.01) (Figure 3A). In a model of acute pneumonia (2–5×10^7^ cfu) 502A displayed increased virulence as compared to USA300 with an average of 1.2×10^5^ cfu in BALF compared to 3.2×10^2^ USA300. This increased bacterial burden was also evident in lung tissue, 502A counts were 5-fold higher than USA300 (Figure 3B). Indicative of the high bacterial counts, 502A infected mice had higher (978 vs 547 µg/ml, P<0.001) protein content in their BALF, an indicator of lung injury (Figure 3C). There were increased numbers of cells recruited to the airway of 502A infected mice, significantly more dendritic cells (82.5% more, P<0.05) and natural killer cells (346%, P<0.01) were recovered from the airways of 502A infected mice (Figure 3D) and significantly greater concentrations of the inflammatory cytokines CXCL1/KC (1043 vs 75 µg/ml, 1290% increased, P<0.001) and IL-6 (986 vs 244 µg/ml, 304% increase, P<0.001) compared to USA300 infected mice (Figure 3E). Levels of IL-1β and TNF were elevated in 502A infected mice, but were not significantly different between the two strains. These results indicate that when the mucosal barrier is bypassed, 502A is able to cause increased inflammation, cytokine expression and lung injury as compared to USA300.

**Figure 4:**
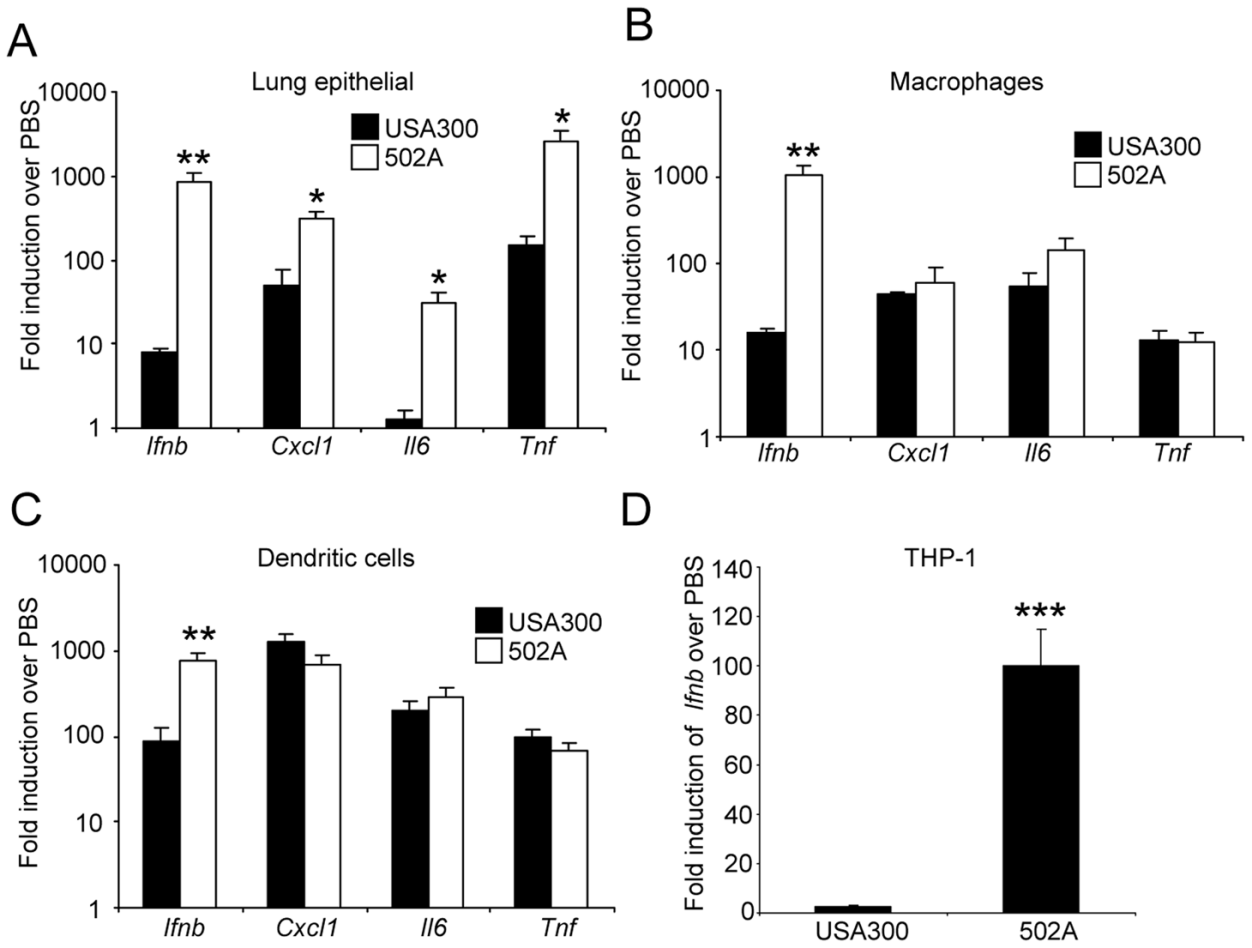
*S. aureus* 502A has enhanced induction of type I IFN signaling. *S. aureus* USA300 and 502A were incubated with A) mouse lung epithelial cells, B) murine bone marrow derived macrophages or C) murine bone marrow derived dendritic cells for 2 h and levels of *Ifnb*, *Cxcl1*(*KC*), *Il6* and *Tnf* determined using qRT-PCR. D) *Ifnb* levels in THP-1 cells after 2 h incubation of *S. aureus* USA300 or 502A. Data are representative of two independent experiments. N = 3. ***P<0.001, **P<0.01 and *P<0.05 compared to USA300.

We hypothesized the in vivo results could be explained by a differential host response. We stimulated mouse lung epithelial cells (Figure 4A), mouse bone marrow derived macrophages (BMM) (Figure 4B) and dendritic cells (BMDC) (Figure 4C) with USA300 and 502A and examined gene expression. Consistent across all three cell types was a substantial increase in type I IFN induction, as indicated by increased *Ifnb* levels. We observed 105-fold, 65-fold and 7.7-fold increases in *Ifnb* induction by 502A compared to USA300 in each of the cells types respectively. In the immune cells, this differential gene induction was limited to *Ifnb* and not classical NF-κB-derived products such as *Cxcl1/KC*, *Il6* or *Tnf* (Figure 4) indicating that the elevated *Ifnb* response was not due to increased bacterial load (Figure S2). We confirmed our mouse cell observations in the human monocytic cell line THP-1 and again observed 502A induces significantly more *Ifnb* (65-fold compared to 2.8-fold) than USA300 (Figure 4D). The increased signaling did not necessarily correlate with bacterial growth (Figure S2). Concomitant with the increased signaling we observed increased in LDH release (Figure S2). These data suggest that the increased virulence in mice may be the result of increased host signaling, namely type I IFN in response to 502A.

### 502A activates a NOD2-IRF5 pathway

**Figure 5:**
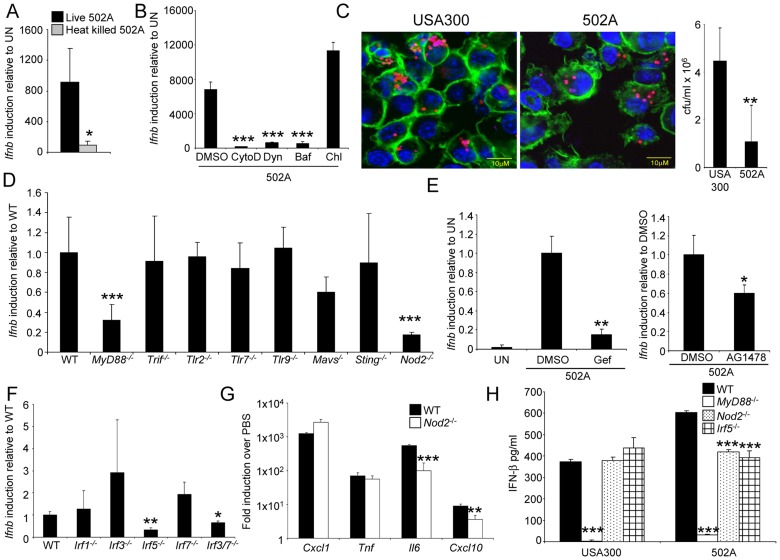
Induction of type I IFN by 502A requires endocytosis, Nod2 and IRF5. BMDC were stimulated by A) live or heat killed 502A, or B) live 502A in the presence of various inhibitors for 2 h before *Ifnb* induction was analyzed by qRT-PCR (n = 3). CytoD-cytochalasin D, Dyn-dynasore, Baf-bafilomycin and Chl-chloroquine. C) pHrodo-labeled *S. aureus* (red) was visualized inside BMDC after 2 h incubation in the presence of AF-488 phalloidin (green) and TO-PRO-3 (blue). Also shown is gentamicin protection assay data from the same experiment (n = 7). D) and F) WT and knockout BMDC were incubated with 502A for 2 h before *Ifnb* levels were assessed using qRT-PCR. E) Induction of *Ifnb* was assessed in WT BMDC in the presence of the RIP2 inhibitor, gefitinib (Gef) or the EGFR inhibitor AG1478. G) WT and *Nod2* ^-/-^ BMDC were incubated for 2 h with 502A before RNA was isolated and gene levels quantitated by qRT-PCR. H) WT and knockout BMDC were incubated with 502A for 24 h before IFN- β levels in the cell supernatant were quantified by ELISA. Data are representative from at least two independent experiments. N = 3. ***P<0.001, **P<0.01 and *P<0.05, compared to WT BMDC infected controls.

USA300 has been shown to activate type I IFN signaling through TLR9 [10]. As type I IFN signaling is activated by bacterial PAMPs that gain access to intracellular receptors, we first documented the ability of 502A to be internalized, then determined the components involved in the pathway. Activation of type I IFN signaling by 502A required live organisms, as heat killed 502A induced 90% less *Ifnb* than live bacteria (Figure 5A), in contrast to USA300 that had no difference in signaling when heat inactivated [10]. The levels of *Ifnb* induction by heat killed 502A were similar to those observed with live USA300 [10].

As dendritic cells are phagocytic, we investigated the role of uptake of 502A as a requirement for type I IFN activation. In the presence of cytochalasin D, an inhibitor of actin polymerization, induction of *Ifnb* was reduced by 97% (P<0.001) (Figure 5B). Inhibition of dynamin-mediated endocytosis was demonstrated by treating cells with dynasore, which inhibits the GTPase activity of dynamin [25] that led to a 91% decrease (P<0.001) in *Ifnb* induction in response to 502A (Figure 5B). In contrast to USA300 [10], *Ifnb* induction by 502A was not inhibited by chloroquine, which indicated a lack of endosomal TLR involvement. Induction was inhibited by bafilomycin that inhibits the vacuolar H^+^ATPase, V-ATPase [26] (93% reduction, P<0.001) (Figure 5B). Cellular uptake was confirmed by the visualization of 502A as well as USA300 inside BMDC. *S. aureus* were labeled with a pH indicator dye, such that only those cells inside acidic compartments fluoresced red (Figure 5C) however, we did not observe any major differences in intracellular location between the strains. By 2 hours we did observe reduced numbers of 502A inside BMDC compared to USA300 (Figure 5C).

Based on our observations that uptake and processing were required for activation of type I IFN by 502A we examined several intracellular and cytosolic adaptor and receptor proteins associated with induction of type I IFN. In addition to an involvement of MyD88, we observed that NOD2 was also required (Figure 5D). As opposed to USA300, TLR9 was not involved in sensing of 502A [10]. Cells lacking NOD2 induced 83% less *Ifnb* (P<0.001) in response to 502A. We further confirmed the involvement of NOD2 by inhibiting its downstream kinase RIP2 with the inhibitor gefitinib [27] (85% reduction, P<0.01) (Figure 5E). As gefitinib can also inhibit EGFR we showed that AG1478, a specific EGFR inhibitor did not influence *Ifnb* induction to the same degree as gefitinib (Figure 5E).

The involvement of NOD2 in type I IFN activation is not fully characterized [28]–[30]. We screened BMDC from mice lacking several different interferon regulatory factors (IRF) for *Ifnb* induction in response to 502A and identified IRF5 as being the major IRF associated with induction by 502A. BMDC lacking IRF5 had a 67% decrease in *Ifnb* induction. In addition we also observed that mice lacking both IRF3 and IRF7 had 35% less (P<0.05) *Ifnb* induction (Figure 5F). To determine the effects of NOD2-mediated signaling we compared levels of gene transcript between WT and *Nod2* ^-/-^ BMDC. *Nod2* ^-/-^ BMDC did not express reduced levels of *Cxcl1/KC* or *Tnf*, but did express significantly reduced levels of *Il6* (82% reduction, P<0.001) and *Cxcl10* (60% reduction, P<0.01), gene products known to be influenced by type I IFN (Figure 5G). Consistent with our RNA data, significantly reduced levels of IFN-β were detected in *MyD88* ^-/-^, *Nod2* ^-/-^ and *Irf5* ^-/-^ BMDC. Levels of 502A induced IFN-β were reduced by 30% (P<0.001) and 35% (P<0.001) in *Nod2* ^-/-^ and *Irf5* ^-/-^ BMDC respectively compared to WT cells. IFN-β in *Nod2* ^-/-^ and *Irf5* ^-/-^ cells in response to 502A and USA300 were equivalent (Figure 5H). Thus the increased type I IFN response by 502A appears to be the result of signaling via NOD2 and IRF5 and suggests that specific staphylococcal strains can activate different mechanisms of sensing and activation of type I IFN signaling.

### Increased 502A autolysis correlates with IFN-β induction

**Figure 6:**
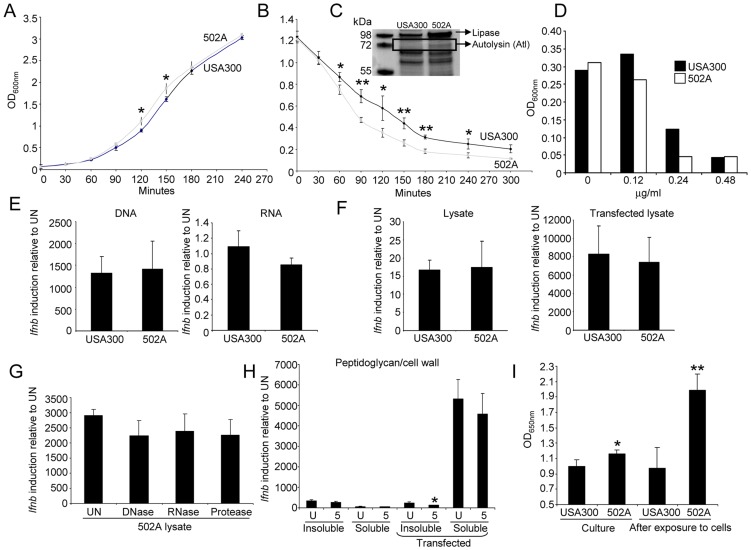
502A has enhanced autolysis. A) Growth curve of USA300 and 502A. Data is an average of three independent experiments. B) Autolysis assay of USA300 and 502A. Black line-USA300, grey line-502A. Data is an average of three independent experiments. C) Coomassie stained polyacrylamide gel of concentrated secreted proteins from USA300 and 502A. Proteins identified by mass spectrometry are indicated. D) Inhibition of growth of *S. aureus* by lysostaphin. Shown is a representative of three independent experiments. E) Purified DNA and RNA from USA300 and 502A was transfected into BMDC and 6 h later *Ifnb* induction was assessed by qRT-PCR. F) Bacterial lysates were generated by sonication and incubated in the presence or absence of transfection reagent for 6 h with BMDC. *Ifnb* levels were then assessed using qRT-PCR. G) Lysates of 502A were generated using sonication. Lysates were then left untreated (UN) or treated with DNase, RNase or protease before stimulating BMDC and then assessing levels of *Ifnb* by qRT-PCR. H) Peptidoglycan was isolated from strains and kept as insoluble or digested to produce soluble peptidoglycan. Samples were incubated in the presence or absence of transfection reagent for 6 h on BMDC before *Ifnb* was assessed by qRT-PCR. I) Peptidogylcan quantification from clarified exponential phase cultures and HaCat supernatant after bacterial exposure. Data are representative of two independent experiments (n = 3). **P<0.01 and *P<0.05, compared to USA300.

NOD2 recognizes muramyl dipeptide of peptidoglycan, sampling peptidoglycan from whole as well as lysed bacteria in the cytosol [31], [32]. Accordingly we postulated that 502A might be more autolytic thus providing increased PAMPs to activate the host response. We first examined the growth rates of 502A and USA300 and consistently observed a faster exponential growth phase by 502A (Figure 6A). 502A also displays significantly greater autolysis compared to USA300 in a triton X-100 autolysis assay (Figure 6B). Increased amounts of the major staphylococcal autolysin, Atl, was present in the secreted fraction of cultures as identified by mass spectrometry (Figure 6C; controls Figure S3). Increased autolysis was further suggested by the susceptibility of 502A to lysostaphin (Figure 6D), an endopeptidase that specifically targets peptidoglycan in the cell wall of staphylococci [33] as well as increased susceptibility to cell wall directed antibiotics (data not shown).

The contribution of other PAMPs to induction of *Ifnb* was evaluated. Purified DNA, RNA and lysates of USA300 and 502A, or 502A lysates treated with DNase, RNase and protease elicited no significant differences in *Ifnb* induction and also between the two strains (Figure 6E–G). We also examined purified peptidoglycan from both strains in two forms, insoluble and soluble (digested with mutanolysin and lysostaphin overnight). Insoluble peptidoglycan gave equivalent low levels of induction regardless of the presence of transfection reagent. Soluble peptidoglycan, upon transfection, induced a 5000-fold increased in *Ifnb* (Figure 6H), much more than other agonists however, no differences were observed between strains. As autolysis should release cell contents we quantified peptidoglycan levels from cultures. Exponential phase cultures of 502A had significantly (16%, P<0.05) higher levels of peptidoglycan compared to USA300 (Figure 6I). After interaction with host cells, we saw an even greater difference with a 100% increase (P<0.01) in peptidoglycan in 502A treated cells compared to USA300 treated (Figure 6I). These observations suggest that the enhanced autolysis of 502A releases highly stimulatory peptidoglycan providing greater NOD2 stimulation than USA300 to activate the type I IFN pathway.

### Type I IFN signaling contributes to the pathogenicity of 502A

**Figure 7:**
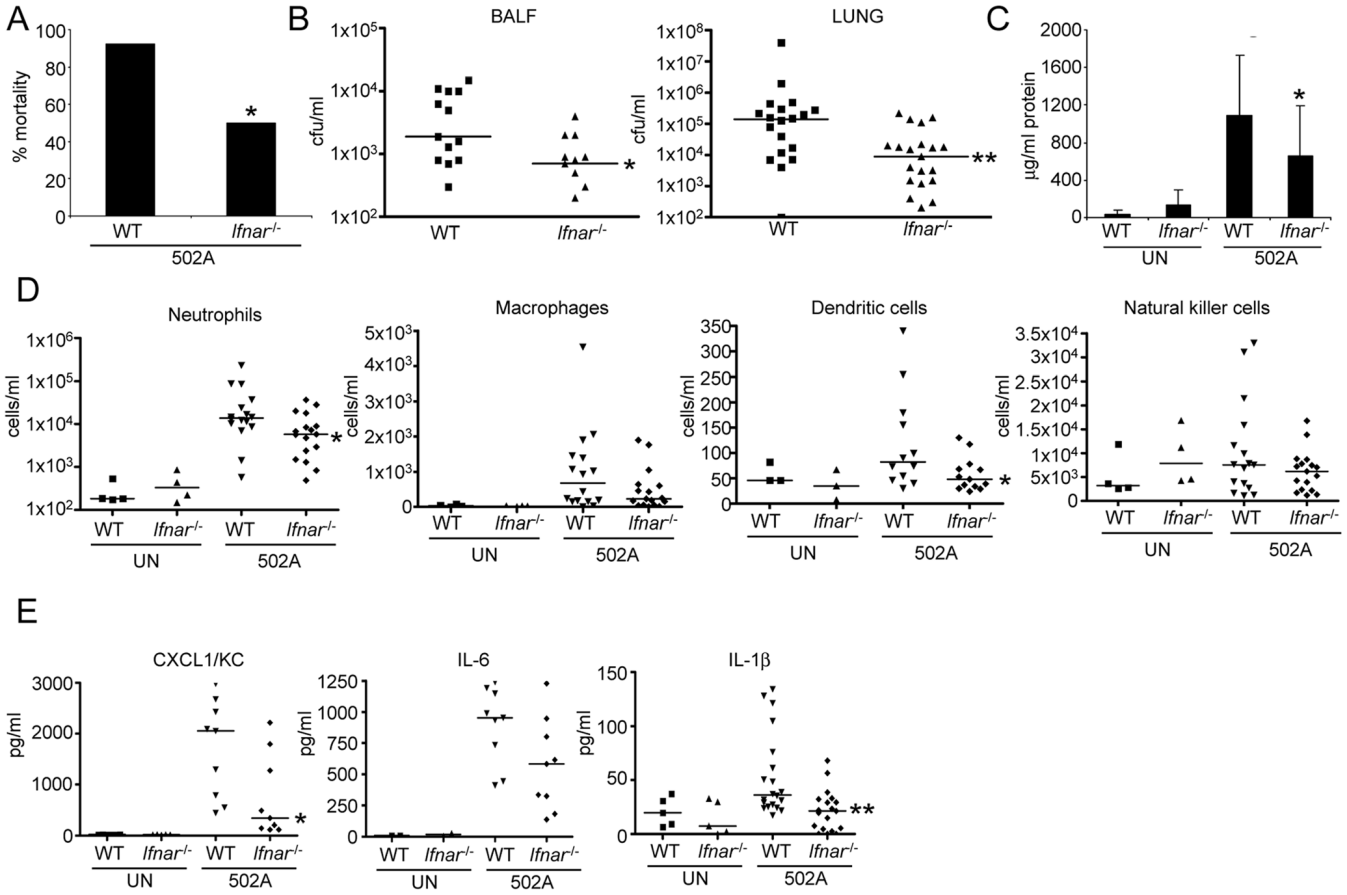
Type I IFN signaling contributes to in vivo virulence of 502A. A) WT C57Bl/6J and *Ifnar* ^-/-^ mice were infected with 10^8^ cfu of 502A for 20 h and mortality assessed (n = WT-13, *Ifnar* ^-/-^-14). WT C57Bl/6J and *Ifnar* ^-/-^ mice were infected with 10^7^ cfu of 502A for 20 h. B) BALF and lung homogenate were assessed for bacterial numbers. C) Total protein content in BALF (n = WT-19, *Ifnar* ^-/-^-20). D) Cells in BALF were stained with fluorescent antibodies and analyzed by flow cytometry. E) Cytokines were measured by ELISA from BALF. Data is from at least two independent experiments. Each point represents a mouse. Lines display median values. ***P<0.001, **P<0.01 and *P<0.05, compared to WT infected controls.

**Figure 8:**
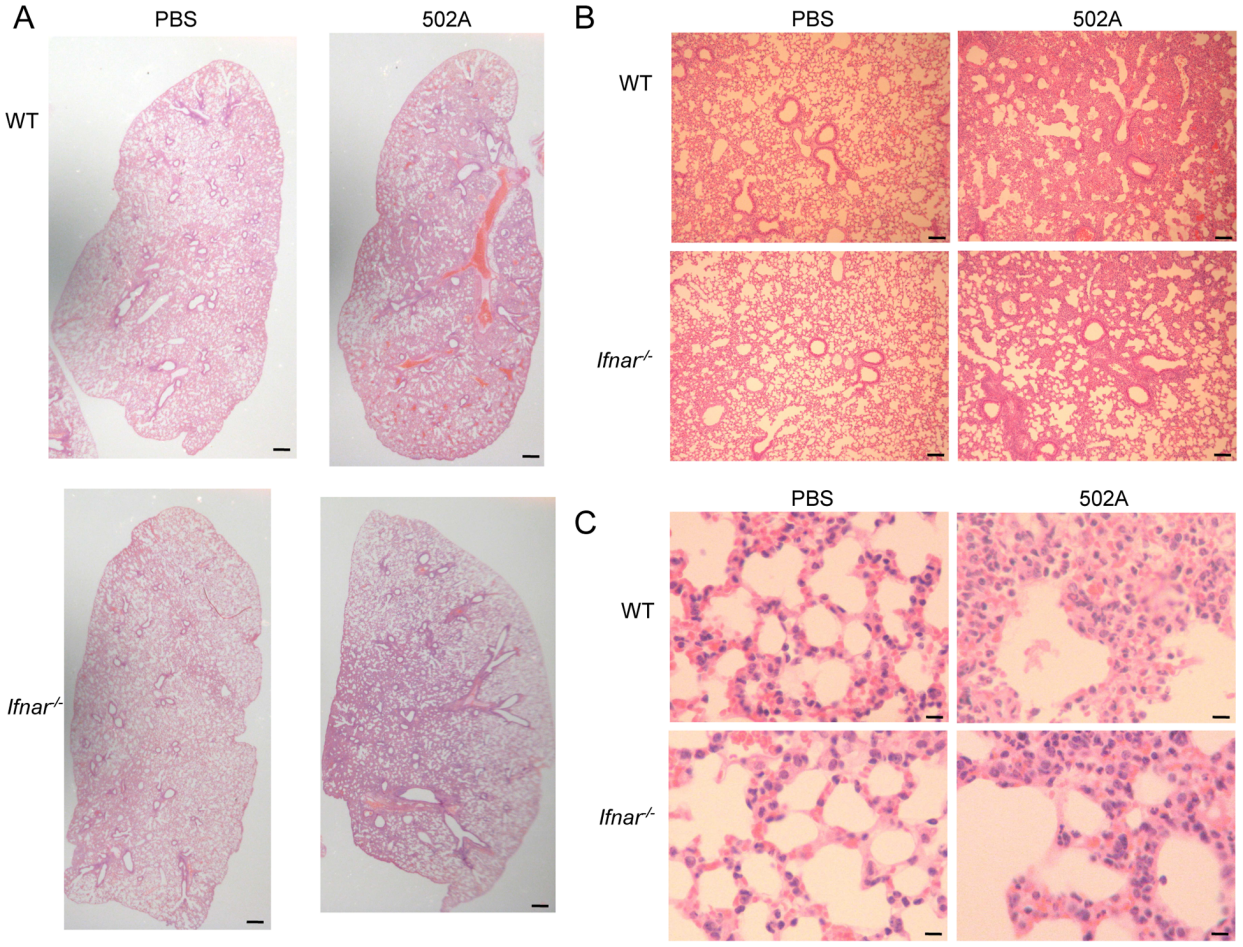
Type I IFN signaling contributes to pulmonary pathology in response to *S. aureus* 502A. WT C57Bl/6J and *Ifnar* ^-/-^ mice were infected with 10^7^ cfu of 502A for 20 h. H&E sections of lung tissue at A) 20× (scale bar equals 350 µm), B) 100× magnification (scale bar equals 100 µm) and C) 400× magnification (scale bar equals 25 µm).

To determine the contribution of type I IFN signaling to the host response by 502A we compared WT and *Ifnar* ^-/-^ mice lacking the type I IFN receptor, in our model of murine acute pneumonia. At a higher inoculum of bacteria we observed a significant decrease in mouse mortality. Only 50% of *Ifnar* ^-/-^ mice succumbed to infection compared to 92% of WT mice (P<0.05) (Figure 7A). When mice were intranasally infected with a lower inoculum we observed a 5.2-fold reduction in bacterial burden in the BALF (P<0.05) and 61-fold less bacteria in lung tissue (P<0.01) (Figure 7B). This decrease in bacterial burden in the airway was also evident in diminished lung damage. Total protein content in BALF, an indicator of injury was reduced by 40% (P<0.05) in 502A infected *Ifnar* ^-/-^ compared to WT infected animals (Figure 7C). We also observed reductions in immune cell recruitment to the airway in *Ifnar* ^-/-^ infected mice. Neutrophils were reduced by 74% in *Ifnar* ^-/-^ mice (WT-3.6×10^4^/ml, *Ifnar* ^-/-^-9.4×10^3^/ml; P<0.05) and dendritic cells were reduced by 52% (WT-120 cells/ml, *Ifnar* ^-/-^-58 cells/ml; P<0.05) (Figure 7D). These reductions were a likely reflection of the reduced bacterial burden. As a further indicator of the host response to the infection we quantitated cytokine levels in the BALF. We observed significant decreases in both CXCL1/KC and IL-1β levels (Figure 7E). CXCL1 levels were reduced by 56% in *Ifnar* ^-/-^ infected mice compared to WT infected animals (1701 vs 747 pg/ml; P<0.05) while IL-1β levels were reduced by 60% (53 pg/ml vs 21 pg/ml; P<0.01) levels not significantly different from uninfected mice (Figure 7E). The cumulative effect of the reduced bacterial burden and immune response was illustrated in H&E stained lung sections. These showed that in response to 502A, WT mice exhibited increased consolidation, cellular infiltrate and loss of alveolar architecture as compared to the *Ifnar* ^-/-^ mice (Figure 8). Thus, the induction of type I IFN signaling by 502A contributes significantly to the virulence of this organism in a model of pneumonia.

## Discussion

*S. aureus* infections are a tremendous cause of human morbidity and mortality. Despite the intense interest in their epidemiology and mechanisms of pathogenesis, very basic questions regarding their virulence remain unsolved. The participation of innate immune signaling in the prevention and clearance of staphylococcal infection is established however, the contribution of host signaling to the pathology elicited by invasive *S. aureus* has not. In *S. aureus* pathogenesis much of the focus is on virulence factors and while USA300 had an invasive advantage over 502A, 502A had greater pathogenic propensity in our model of acute murine pneumonia. This comparison of two well characterized strains of *S. aureus* associated with human colonization and infection confirms, as often observed in human staphylococcal pneumonia, that the intensity of the inflammatory response induced in the lung correlates with tissue damage and poor outcome. Moreover, we suggest that staphylococcal induction of the type I IFN cascade is responsible for this increased pathogenicity. Once across the mucosal barrier equivalent inocula of 502A caused significantly more disease than the comparator strain USA300 by all of the conventional standards. It caused greater mortality, was less efficiently cleared from airway and lung tissue, induced greater destruction of the lung and invoked a significantly greater proinflammatory response as defined by cytokine levels and the influx of immune cells. We have demonstrated that signaling through NOD2 and IRF5 by strain 502A in contrast to TLR9 and IRF1 by USA300 [10] generates an exuberant type I IFN response and that this type I IFN response is the predominant factor associated with the induction of pathology and mortality in a murine model of infection.

The potential of 502A for virulence was well appreciated in the past, as it was a standard strain used in numerous assays of phagocytosis and models of infection [34]–[36]. Yet, except for a very few notable exceptions, it was quite innocuous when used to colonize thousands of newborn infants in the 1950's [14]–[17].

Our studies identified that the ability of USA300 to invade, and cross keratinocyte and epithelial cells makes it a virulent organism, particularly since 502A appears to encode a similar genetic repertoire of virulence factors. One prominent virulence factor missing from 502A was the Panton-Valentine toxin or PVL however, we have shown previously that it is not involved in keratinocyte or epithelial invasion [37], [38]. Another factor enhancing the virulence of USA300 is its ability to survive the antimicrobial activity of the mucosal barrier. 502A was significantly more susceptible to products produced by both skin and airway cells, therefore the increased invasiveness of USA300 may be a result of its resistance to host antimicrobial killing. In the acute phase 502A induced greater signaling and release of LDH by cells, but over time its increased capacity to undergo autolysis and susceptibility to host products led to reduced persistence in vitro. Nonetheless, 502A displayed increased virulence in our murine model of pneumonia that was dependent on its activation of the type I interferon cascade. We have observed with *S. aureus* USA300 that type I IFN signaling is involved in bacterial clearance and observed a slight reduction in TNF production [10]. The ability of 502A to induce significantly higher levels of type I IFN allows us to observe with better clarity the role this pathway plays in staphylococcal infection. Thus, despite a relatively impaired ability to invade either locally or systemically, 502A was able to induce a robust inflammatory response that caused greater damage than that attributed to USA300.

Genomic analysis of 502A as compared with USA300 suggests an explanation for these observations. The presence of the ACME element and the spermine/spermidine acetyltransferase SpeG in USA300, but not 502A, and its association with the ability to withstand the antimicrobial activity of spermidine and spermine on human skin could well account for the relative success of USA300 to avoid epithelial and keratinocyte killing [23], [39]. We did observe a decrease in protein A production in 502A however, we have shown this factor to not play a role in type I IFN signaling in immune cells and would expect an increase in expression if we were to attribute this factor to the enhanced virulence of 502A. While our genomic analysis did not discover many differences there were several different secreted protein bands observed between USA300 and 502A that may account for their phenotypic differences. The conservation of other major classes of virulence factors, the phenol soluble modulins, α-toxin, as well as other toxins, with the exception of PVL, suggests that these well accepted virulence factors were not responsible for the enhanced tissue destruction associated with 502A pneumonia and further illustrates the role the host response plays in pathogenesis. Given the increased susceptibility of 502A to mucosal clearance, its enhanced virulence when delivered intranasally in the murine model is even more striking.

Phenotypic comparison of 502A and USA300 suggests the increased propensity of 502A to undergo autolysis is a reason for its heightened pathogenicity. The ability of the released cell wall fragments, namely peptidoglycan, to activate cytosolic Nod2 and IRF5 signaling resulted in a significantly increased induction of *Ifnb*, a pathway once previously described in response to *Mycobacterium tuberculosis* [29]. It is also interesting to compare the location of the signaling activated by each strain. USA300 signals via TLR9, which is located in the endosome, while 502A signals via the cytosol. One avenue of future investigation will be to determine the processing differences between these strains. A penicillin susceptible strain, 502A had an increased rate of exponential growth, increased production of Atl, a major autolysin, increased spontaneous autolysis, peptidoglycan release and corresponding increased susceptibility to cell wall active antimicrobial. These results suggest that autolysis plays an important role in the release of peptidoglycan leading to enhanced type I IFN signaling by 502A. Consistent with the involvement of NOD2, autolysins aid in cell division through degradation of peptidoglycan, the ligand for NOD2 [40]. Of note, the induction of NOD2 and IRF5 appears to be specific to 502A, as previous studies of *Ifnb* induction by USA300 demonstrated that signaling involved staphylococcal DNA activation of TLR9 via IRF1 [10], an endosomal process in contrast to the cytosolic NOD2 pathway observed with 502A. The involvement of NOD2 signaling in the intensity of inflammatory responses is not unexpected as polymorphisms in NOD2 are closely linked to the pathogenesis of inflammatory bowel disease, another example of a pathological response to mucosal bacteria [41]–[43].

We also observed other differences in signaling activated by the two staphylococcal strains. There was some involvement of IRF3/7 at the RNA level for induction of *Ifnb*, which were associated with significant reductions in protein levels. MyD88 was also involved in *Ifnb* signaling, with a significant decrease in *Ifnb* RNA causing a significant drop in IFN-β. MyD88 may also influence post-transcriptional events in protein production. The ability of two strains of the same pathogen to activate distinct host innate immune responses further illustrates the degree of adaptation of the pathogen to the host as well as redundancy in the immune system to sense the same species through multiple mechanisms.

Our approach using two disparate strains to understand the pathogenesis of *S. aureus* pneumonia is in contrast to most other studies that use isogenic mutants to query the nature of the host response. Our data suggest that the intensity of innate immune signaling, and in this case, the type I IFN cascade, correlates significantly with the outcome of acute infection. The finding that 502A, used in high dose to inoculate neonates, was highly virulent was entirely unexpected, particularly in comparison to USA300, in which nasal colonization and subsequent invasive infection are closely associated [4], [5]. Our results suggest that the prevalence of USA300 in invasive infection is due, in large part, to its ability to persist on skin and mucosal surfaces. Once across the mucosal or epithelial barrier, the immunostimulatory potential of the “non-pathogenic” 502A enables this organism to evoke damaging host immune responses.

Staphylococcal virulence, not unexpectedly, is multifactorial and involves the relative ability of individual *S. aureus* strains to activate type I IFN signaling, once they have crossed the mucosal barrier. The participation of the host innate immune response to specific pathogens is a key determinant in the outcome of infection.

## Materials and Methods

### Bacterial strains and cell culture

*S. aureus* USA300 FPR3757 [2] and 502A [16] were grown in Luria Bertani broth at 37°C. Heat-killed preparations of *S. aureus* were obtained by heating cells at 65°C for 1.5 h. Minimal inhibitory concentrations using lysostaphin (Sigma-Aldrich) were conducted in microtitre plates for 16 h.

To identify differential protein expression overnight cultures of *S. aureus* were grown, pelleted and culture supernatant concentrated 20× using Amicon 10 kDa columns. Samples were run on SDS-PAGE, bands excised, digested and identified using LC-MS spectroscopy and Mascot protein identification software (Columbia University Medical Center Protein Core).

The human keratinocyte cell line HaCat, human bronchial epithelial line16HBE, mouse BMDC and primary human keratinocyte cells were grown as described before [38], [44], [45]. RPMI 2650 human nasal epithelial cells were grown in minimal essential medium with 10% heat inactivated fetal bovine serum with penicillin and streptomycin. Murine lung epithelial cells (MLE) LA4 were grown in F-12K Kaighn's modification media with serum and antibiotics as above. Transmigration of *S. aureus* across polarized 16HBE airway epithelial cells was performed as before [37], [44]. The human monocyte line THP-1 was grown in RPMI 1640 (life) with 10% heat inactivated fetal bovine serum with penicillin and streptomycin. Cells were differentiated by the presence of 10 nM phorbol-12-myristate-13-acetate for 24 h. Stimulations with THP-1 cells were at an MOI of 10. Gentamicin protection assays were performed using an MOI of 25 for 2 h before cells were washed and 400 µg/ml of gentamicin was incubated for 2 h before bacteria were serially diluted. For uptake assays *S. aureus* was labeled with fluorescein as before [10] and added for the times indicated before HaCat cells were washed and analyzed by flow cytometry. Cytotoxicity [lactate dehydrogenase (LDH)] was measured using the cytotoxicity detection kit (Roche) according to the manufacturer's instructions.

BMDC were stimulated with *S. aureus* (MOI 100) for 2 h for RNA experiments and 24 h (MOI 10) for cytokine analysis. Staphylococcal lysate experiments were performed using 1×10^8^ inactivated cfu as before [45]. Experiments with cellular inhibitors were performed by preincubating the cells for 30 min to 1 h prior to bacterial stimulation using: cytochalasin D 20 µM (Sigma), dynasore 80 µM [25], chloroquine 10 µM, bafilomycin (Invivogen) 1 µM, gefitinib 10 µM (Invivogen) and AG1478 10 µM (Calbiochem). Transfection of BMDC was typically performed using 1 µg of nucleic acid and 5 µg of peptidoglycan using FuGene (Roche). DNA was extracted using the DNeasy kit (Qiagen) after lysing *S. aureus* with 4 U/ml mutanolysin, 10 µg/ml lysostaphin and 25 mg/ml lysozyme for 1 h at 37°C.

### Immunoblotting

Exponential phase cultures (10 ml of OD600 nm 1.0) were resuspended in EDTA-free protease inhibitor (Roche) with extraction buffer (30% w/c raffinose, 50 mM Tris-HCl pH 7.5, 20 mM MgCl_2_ and 100 mg/ml lysostaphin) and incubated at 37°C for 8 min. Clarified supernatant was mixed with Laemmli sample bugger with β-mercaptoethanol and boiled for 10 min. Immunoblotting was performed with rabbit anti-α-toxin antibody (1∶5000) and anti staphylococcal antibody (1∶5000) (704; Santa Cruz Biotechnology). Densitometry was performed using ImageJ (1.45S).

### Genome sequencing and analysis

Genomic DNA was prepared from overnight cultures of 502A strain (RN6607 BK21236 NRS149) using the Tissue DNeasy kit (Qiagen) as above. Library preparation and sequencing was done using the Ion Torrent Personal Genome Machine 316 chip, performed by the Columbia Genome Center using manufacturer-specified protocols. Sequence assembly was performed with Newbler (http://454.com/products/analysis-software/index.asp), generating 181 total contigs (N50 = 25, median contig = 2955). The total size of the contigs was 2,740,204 bp. Preliminary Annotation was done using the Rapid Annotation using Subsystem Technology (RAST) server (http://rast.nmpdr.org). We focused our initial analysis on known *S. aureus* virulence factors. To create a heat map representation of selected genes we used BLAST-blast analysis (http://blast.ncbi.nlm.nih.gov) using default settings on the megablast algorithm and surveying the nr/nt databases. Query sequences were from the USA300 TCH1516 genome sequence or the 502A sequence as noted.

Selected *S. aureus* genomes were used to create a whole genome phylogenetic matrix of gene coding regions using the Ortholog ID process [46]. The matrix was further pruned to include only genes found in more than 96% of known *S. aureus* genomes, and includes only variable nucleotide positions. The final matrix had 93,249 characters. Phylogenies were constructed using PAUP 4b10 (http://paup.csit.fsu.edu) using the maximum parsimony criterion with random addition (RA) followed by tree bisection and reconnection (TBR), Nreps = 1000, with characters and character state transformations given equal weight. Node support was calculated based on 1000 bootstrap pseudoreplicates [47] using the heuristic search strategy above.

### Peptidoglycan isolation

Isolation of peptidoglycan was performed on 100 ml of exponential phase culture. Cells were washed in saline before boiling for 30 min in 4% SDS and left to stand overnight at room temperature. The next day cells were boiled for a further 15 min, left to cool for 1 h before centrifugation at 14,000×*g* for 15 min. After washing in water the extract was treated with 2 mg/ml RNase, 2 mg/ml DNase and 10 mg/ml trypsin before washing to obtain the insoluble fraction. Soluble peptidoglycan was obtained by treating the sample with 10 µg/ml mutanolysin and 100 µg/ml lysostaphin overnight at 37°C. Peptidoglycan was quantified from clarified cultures using the silkworm larvae plasma (SLP) reagent set (Wako) according to the manufacturer's instructions.

### Autolysis assay

Triton X-100 autolysis assays were performed as described elsewhere [48].

### Mice studies

C57Bl/6J, *Tlr2* ^-/-^, *Mavs* ^-/-^, Nod2^-/-^ and *Trif* ^-/-^ and mice were from Jackson Laboratories, and *Tlr7* ^-/-^ mice were from Regeneron. Mice were intranasally infected with 2–5×10^7^ cfu of *S. aureus* for pneumonia studies, or 7×10^8^ cfu for mortality for 20 h as previously described [10]. Protein content in bronchoalveolar lavage fluid (BALF) was measured using Bradford protein assay. Staining cells for flow cytometry has been described elsewhere [45]. Cells were labeled with combinations of fluorescein isothiocyanate-labelled anti-Ly-6G (Gr-1; RB6-8C5; Biolegend), PerCP-Cy5.5-labelled anti-CD11c (N418; Biolegend), allophycocyanin-labelled anti-MHC II (I-A/I-E; Biolegend) and phycoerythrin-labelled anti-NK 1.1 (NKR-P1C, Ly-55; Biolegend). Neutrophils were defined as Ly6G^+^/MHCII^−^, macrophages as CD11C^+^/MHCII^low-mid^ and dendritic cells as CD11c^+^/MHCII^high^. Data were analyzed using WinMDI (version 2.8; Joseph Trotter).

### RNA analysis

RNA was isolated using the eZNA Total RNA kit (Omega bio-tek) followed by DNase treatment using DNAfree (Life Technologies). cDNA was synthesized using the High Capacity cDNA Reverse Transcription Kit (Applied Biosystems). qRT-PCR was performed using Power SYBR Green PCR Master Mix (Applied Biosystems) in a StepOne Plus thermal cycler (Applied Biosystems). Samples were normalized to β-actin. Primers for mouse actin, *Ifnb*, *Cxcl1, Cxcl10* and *Il6* have been described elsewhere [10], [45].

### Cytokines

Cytokine levels were quantified using ELISA to IFN-β (PBL Interferon), CXCL1/KC (R&D Systems), IL-6 (R&D Systems), IL-1β (eBioscience) and TNF (eBioscience).

### Microscopy

Mouse lungs were fixed overnight in 4% paraformaldehyde before being embedded in paraffin. Slides of paraffin sections were stained with hematoxylin and eosin (H&E) and imaged on an Olympus Cx41 microscope and images captured using a Canon Powershot S5IS camera.

BMDC were incubated with exponential phase bacteria labeled with 1 mM of pHrodo (Life Technologies) for 2 h. Fluorescein isothiocyanate phalloidin (Life Technologies) and TO-PRO3 (Life Technologies) were incubated in the presence of 0.2% Triton X-100 prior to fixing in 4% paraformaldehyde and visualization with a Zeiss LSM 510 META scanning confocal microscope

### Statistics

Significance of data that followed a normal distribution was determined using a two-tailed Student *t* test, and for data that did not follow a normal distribution, a nonparametric Mann-Whitney test was used. Dichotomous survival was assessed using the Fisher's exact test. Statistical outliers were identified using Grubbs' test and removed. Statistics were performed with Prism software (GraphPad, La Jolla LA USA).

### Ethics statement

Animal work in this study was carried out in strict accordance with the recommendations in the Guide for the Care and Use of Laboratory Animals of the National Institutes of Health, the Animal Welfare Act and US federal law. The protocol was approved by the Institutional Animal Care and Use Committee (IACUC) of Columbia University (protocol AAAC3059).

## Supporting Information

Figure S1 **Comparison of protein A and α-toxin production.** Clarified cell lysates were probed with anti-staphylococcal antibody to detect protein A and supernatants from overnight cultures were blotted for α-toxin. Densitometry compared to USA300 is provided underneath blots. Protein stained (Ponceau S) membranes are shown for loading controls.
(TIF)

Figure S2 **Comparison of USA300 and 502A in growth and cytotoxicity of cells.** Exponential phase USA300 and 502A were incubated with A) HaCat, B) THP-1, C) LA-4 and D) BMDC cells for 2 h before bacteria were quantified and clarified supernatants tested for LDH activity. ***P<0.001, **P<0.01 and *P<0.05, compared to WT levels.
(TIF)

Figure S3 **Comparison of secreted proteins between USA300 and 502A.** Strains were grown to either A) exponential or B) stationary phase, cultures clarified, filter sterilized and then concentrated 10× before SDS PAGE analysis and staining with Ponceau S.
(TIF)
