# Supplementary figures and images for: Induction of Type I Interferon Signaling Determines the Relative Pathogenicity of Staphylococcus aureus Strains
Source: PLoS Pathog. 2014 Feb 20;10(2):e1003951. doi: 10.1371/journal.ppat.1003951 (PMC3930619; doi:10.1371/journal.ppat.1003951)

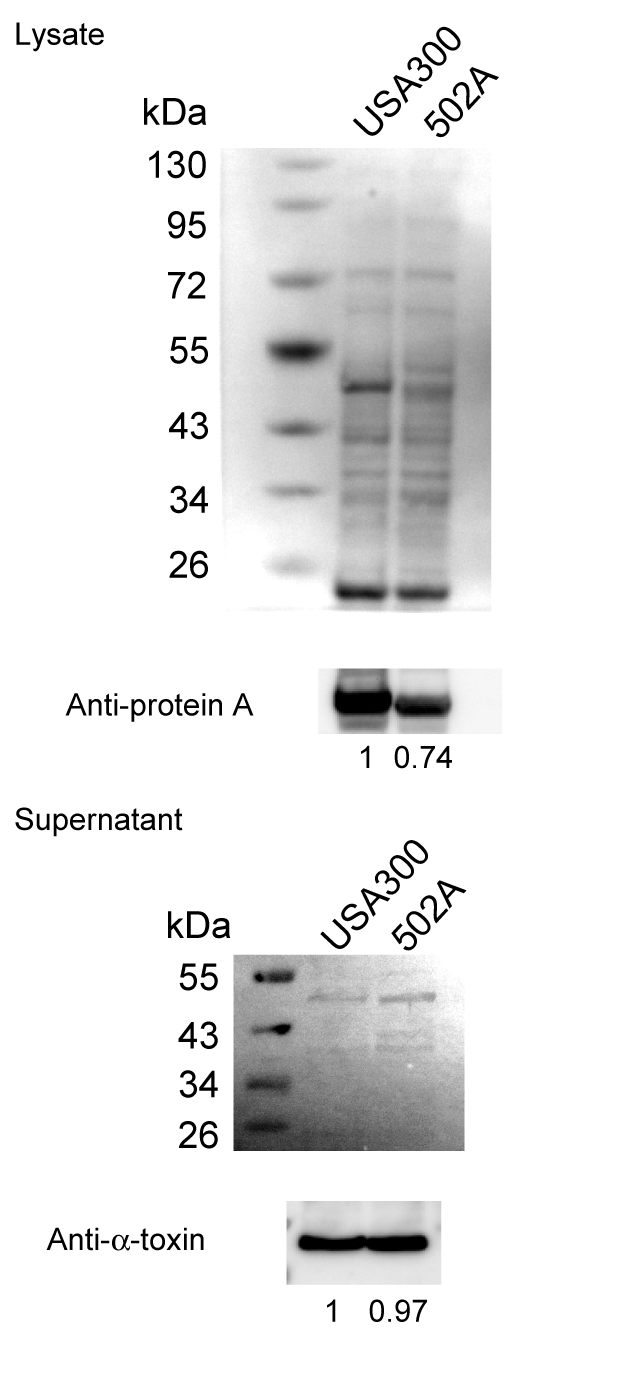

Supplement: Figure S1 — Comparison of protein A and α-toxin production. Clarified cell lysates were probed with anti-staphylococcal antibody to detect protein A and supernatants from overnight cultures were blotted for α-toxin. Densitometry compared to USA300 is provided underneath blots. Protein stained (Ponceau S) membranes are shown for loading controls. (TIF) [file ppat.1003951.s001.tif]

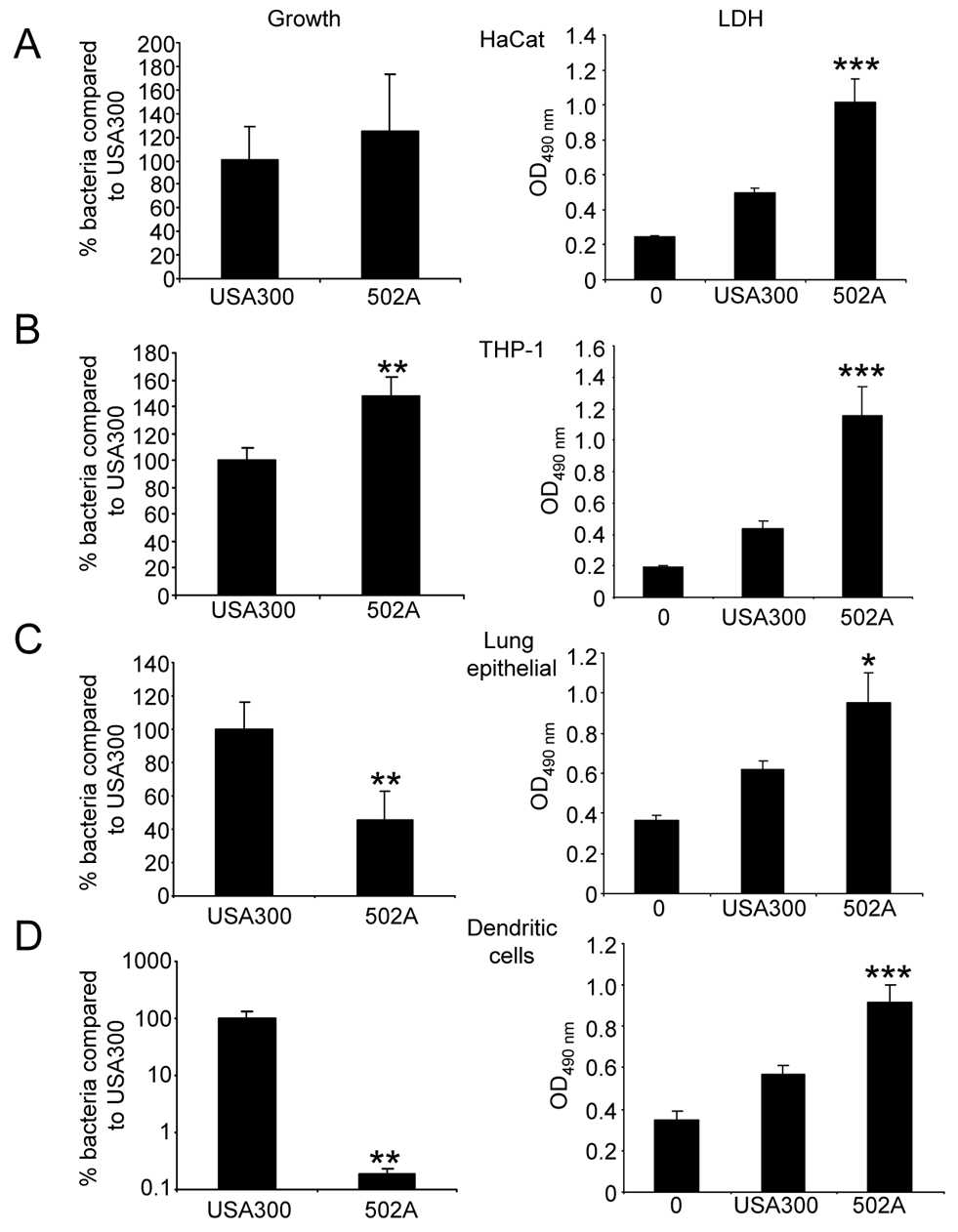

Supplement: Figure S2 — Comparison of USA300 and 502A in growth and cytotoxicity of cells. Exponential phase USA300 and 502A were incubated with A) HaCat, B) THP-1, C) LA-4 and D) BMDC cells for 2 h before bacteria were quantified and clarified supernatants tested for LDH activity. ***P<0.001, **P<0.01 and *P<0.05, compared to WT levels. (TIF) [file ppat.1003951.s002.tif]

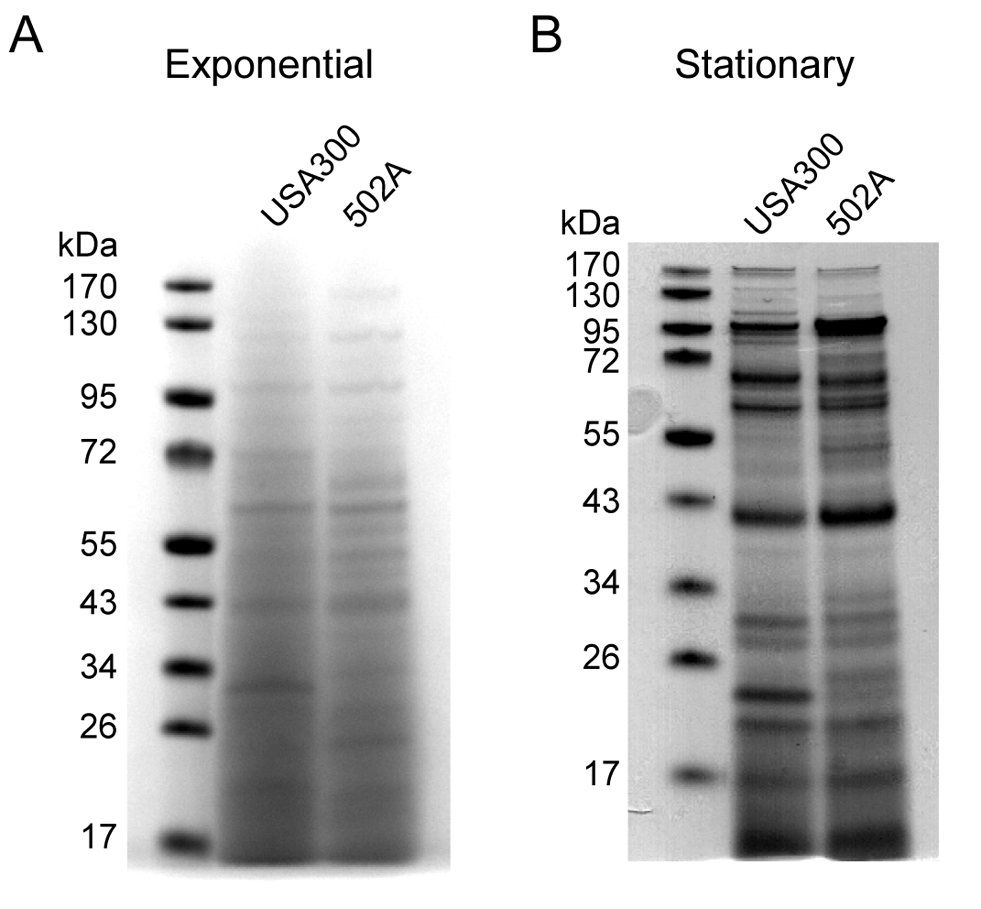

Supplement: Figure S3 — Comparison of secreted proteins between USA300 and 502A. Strains were grown to either A) exponential or B) stationary phase, cultures clarified, filter sterilized and then concentrated 10× before SDS PAGE analysis and staining with Ponceau S. (TIF) [file ppat.1003951.s003.tif]
